# Structural insight for chain selection and stagger control in collagen

**DOI:** 10.1038/srep37831

**Published:** 2016-11-29

**Authors:** Sergei P. Boudko, Hans Peter Bächinger

**Affiliations:** 1 Research Department, Shriners Hospital for Children, Portland, Oregon 97239, USA; 2 Department of Molecular Biology and Biochemistry, Oregon Health and Science University, Portland, Oregon 97239, USA; 3 Department of Nephrology and Hypertension, Vanderbilt University, Nashville, Tennessee 37235, USA

## Abstract

Collagen plays a fundamental role in all known metazoans. In collagens three polypeptides form a unique triple-helical structure with a one-residue stagger to fit every third glycine residue in the inner core without disturbing the poly-proline type II helical conformation of each chain. There are homo- and hetero-trimeric types of collagen consisting of one, two or three distinct chains. Thus there must be mechanisms that control composition and stagger during collagen folding. Here, we uncover the structural basis for both chain selection and stagger formation of a collagen molecule. Three distinct chains (α1, α2 and α3) of the non-collagenous domain 2 (NC2) of type IX collagen are assembled to guide triple-helical sequences in the leading, middle and trailing positions. This unique domain opens the door for generating any fragment of collagen in its native composition and stagger.

Collagen is the most abundant protein in the human body and is the major building block for bone, cartilage, tendon, ligament and skin. Collagen molecules are crucial to tissue organization and physiology with their functions ranging from bulk mechanical strength to delicate instructions to cell receptors.

Every molecule of the 28 types of collagen in humans or other collagen-like proteins contains a collagenous fragment with repeated G-X-Y sequences, where X and Y are any amino acid but often proline and hydroxyproline residues, respectively. Three chains associate with a one-residue shift (stagger, register) to fit glycine residues in the inner core. This type of packing was confirmed by multiple crystal structures of collagen and collagen-like fragments^1^. In the case of hetero-trimeric types of collagen an arbitrary staggering process could generate multiple structurally distinct conformations in the absence of a control mechanism. For example, in type I collagen, which consists of two α1 and one α2 chains, there are three different staggers possible, where α2 chain is placed in the leading, middle or trailing position. In the case of all three different chains the number of different staggers increases to six. Is there a particular type of stagger for each collagen type? Although the answer to this question still remains somewhat unresolved, a number of data tells us about stagger-specific response for ligand binding^2^,^3^,^4^,^5^,^6^ or degradation^7^. In addition, for one collagenous region of type IV collagen the stagger was experimentally determined^8^. If there is a specific stagger in each triple-helical fragment of collagen, then how is it controlled? Mainly two strategies are possible. First, it could be an intrinsic property of the triple helical fragment. This mechanism was confirmed in a number of experiments using several sets of artificial collagen-like sequences, which complement each other by opposite charges, hydrogen bonding or hydrophobicity^9^,^10^,^11^. Whether this mechanism could be also valid for sequences derived from real collagens still needs to be addressed. Second, there is an exogenous predisposition of polypeptide chains that comes from non-triple helical (non-collagenous) regions of the molecule, which makes sterically/energetically possible only one unique stagger.

The collagen repeating sequence has an intrinsic ability to form irregular alignments, which under certain conditions leads to a formation of a gel, also known as gelatin. To avoid such complications every type of collagen has a unique trimerization domain that selects and aligns three specific chains^12^. For most collagen types this domain is located within the C-terminal non-triple-helical domain. For a number of collagen types the atomic structures of the trimerization domain are available. So far four structural classes of collagen trimerization domains are reported: the NC1 domain of type IV collagen^13^,^14^,^15^, the C1q-type domain of types VIII and X^16^,^17^, the multiplexin trimerization domain of types XV and XVIII^18^,^19^ and the C-propeptide of fibrillar collagens (types I, II, III, V and XI)^20^. This structural repertoire should also be extended with an example of a classical α-helical coiled coil domain observed in lung surfactant protein D^21^. In each class, specific regions within the domain are responsible for the formation of homo- or hetero-trimers^12^,^20^. These domains also serve as the nucleus for the zipper-like folding of the triple-helical domain from the C- to the N-terminus of a molecule. However, how the stagger of the triple helix is determined is not clear from the isolated structures of the collagen trimerization domains. Until now no structural information was available on how the triple helical domain is linked to the collagen trimerization domain and whether the triple-helix stagger is determined/influenced by it.

There are other collagens that use different types of trimerization domains. Such domains greatly vary in size, position and structure with the smallest known domain discovered in type IX collagen of only ~35 residues^22^. This domain, NC2 (the second non-collagenous domain, Fig. 1A) was demonstrated to be responsible for stagger control in the adjacent triple helix^23^. Here we report the structural basis of this control.

## Results

**Figure 1:**
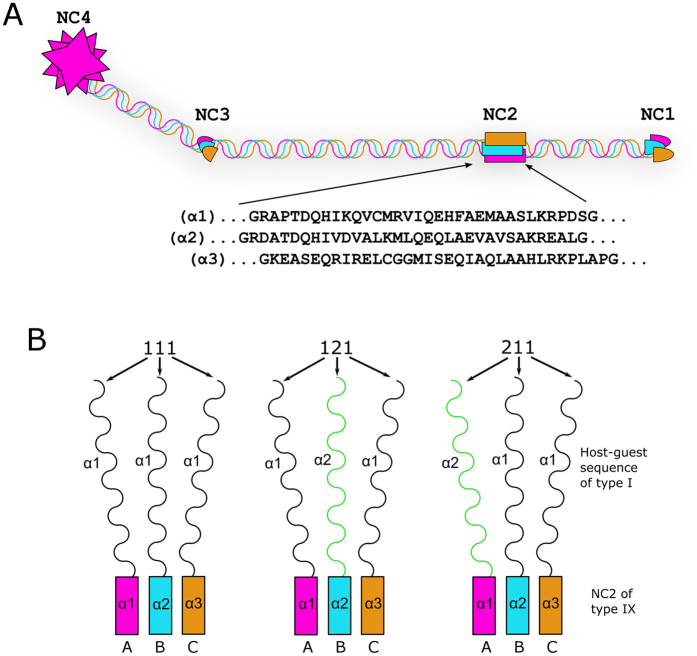
Domain organization of type IX collagen and design of chimeric constructs. (**A**) Four non-collagenous domains (NC1-4) are historically numbered starting from the C-terminus. Sequences of the NC2 domain studied here are shown. (**B**) The three constructs used in this study.

Previously, to test whether the stagger control resides in the NC2 domain we used short native sequences of type I collagen, which is a hetero-trimer of two α1 and one α2 chains. Two host-guest collagen peptides (GPP)_4_-(GXY)_4_-(GPP)_3_, where (GXY)_4_ sequences are from the α1 and α2 chains of human type I collagen, were recombinantly linked to chains A, B and C (corresponding to α1, α2 and α3 in collagen nomenclature and omitted here to avoid confusion) of the NC2 domain in the following combinations designated as α1_A_α1_B_α1_C_ (for short 111), α1_A_α1_B_α2_C_ (112), α1_A_α2_B_α1_C_ (121), α2_A_α1_B_α1_C_ (211) and α2_A_α2_B_α2_C_ (222) (for details see Fig. 1B). The collagenous portion of type I collagen in these complexes formed a stable triple helix, but demonstrated differences in thermal stability and binding affinity to the von Willebrand factor A3 domain^23^.

**Figure 2:**
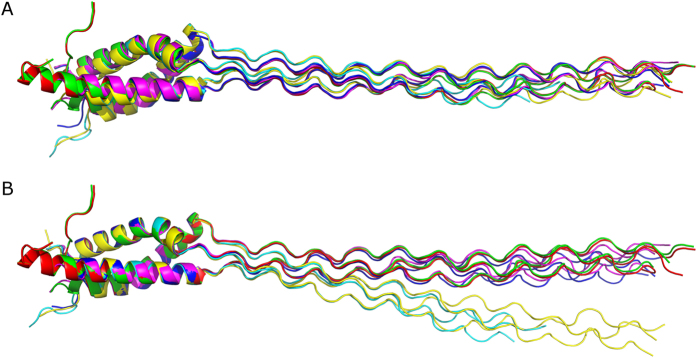
Superimpositions of structures. (**A**) Overall superimposition of six structures (121nat, 121sm, two trimers of 111sm and two trimers of 211sm). (**B**) Superimposition of the same structures within the NC2 domain core.

All five constructs were screened for crystallization, but only three of them yielded crystals of sufficient quality, crystal structures for 111, 211 and 121 were independently solved using the MAD phasing from selenomethionine derivatives (thereafter designated as 111sm, 211sm and 121sm) to 2.25 Å, 2.10 Å and 1.6 Å, respectively (Fig. 2, Table 1). In addition, the structure of 121 (with regular methionines, thereafter designated as 121nat) was solved using molecular replacement to 1.9 Å. Whereas the crystal packing for 111sm and 211sm are similar (two trimers per asymmetric unit), it differs from that for 121nat or 121sm (one trimer per asymmetric unit) (Table 1). In total we obtained six crystal models for the trimer of the NC2 domain and the adjacent triple helix (Fig. 2).

### The structure of the NC2 domain of the hetero-trimeric type IX collagen

**Figure 3:**
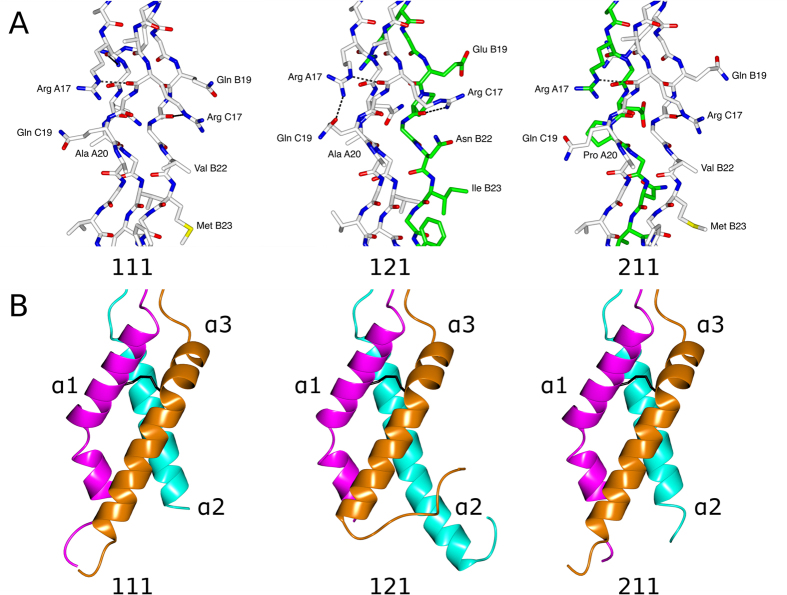
Close-up views. (**A**) The triple helical region of the type I collagen guest sequences. (**B**) The NC2 domain comparison. The disulfide bond between the α1 and α3 chains is shown as cylinders in black. Chain A (α1) – magenta, chain B (α2) – cyan, chain C (α3) – dark orange.

**Figure 4:**
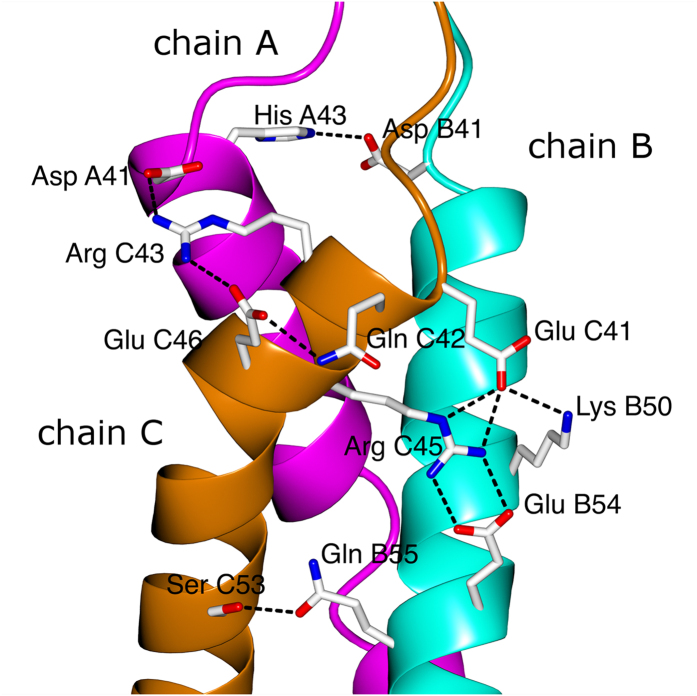
Non-covalent inter-chain bonding within the NC2 domain (shown 121sm). Chain A (α1) – magenta, chain B (α2) – cyan, chain C (α3) – dark orange.

In accord with the secondary structure prediction^22^,^24^, the NC2 domain assumes predominantly an α-helical conformation. Three unique chains form a parallel α-helical right-handed bundle. Whereas the α2 chain contains a single α-helix, α1 and α3 have a short kink and a bend, respectively (Figs 3B and 4). As predicted^22^,^23^, a disulfide bond connects α1 and α3. An overall superimposition of 121nat and 121sm (r.m.s.d. of 0.35 Å) confirmed the identity of the two structures. Overall superimposition of all trimers showed some drastic deviations (Fig. 2A). The most deviated pair is the first trimer (chains A, B, C in the asymmetric unit) of 121sm versus the second trimer (chains D, E, F) of 111sm (r.m.s.d. of 3.01 Å). On the other hand, superimposition within the NC2 domain demonstrated high identity of the trimerization domain and adjacent residues within the triple-helical portion (Fig. 2B). Most of deviations observed for overall superimposition of trimers are attributed to distal flexibility of the triple helical fragments caused by crystal packing (Fig. 2A vs B).

It was suggested that many collagens contain α-helical coiled coil domains that might help in trimerization and stagger formation^25^. The NC2 domain of type IX collagen has been among such domains, but discontinuities in the heptad reapeat pattern (a characteristic feature of the coiled coil) in this domain were pointed out^25^. Although the crystal structure of the NC2 domain demonstrates a high content of α helices in somewhat parallel organization, overall it does not even resemble the coiled coil due to multiple violations of geometry (Fig. 2B). Overall, the NC2 domain structure demonstrates a right-handed bundle of helices as opposed to a left-handed superhelix in classical coiled coils, *e.g.* in lung surfactant protein D^21^. Moreover, the α-helical coiled-coils are normally blunt ended and do not embody a stagger needed to accommodate the triple helix. Nevertheless, the inter-chain interface is stabilized by numerous hydrophobic interactions similar, but not identical to those observed in the coiled coil. In addition a set of hydrogen bonding and ionic interactions contributes to specificity and structural integrity of the trimer (Fig. 4).

### Structure of the triple-helical domain

**Figure 5:**
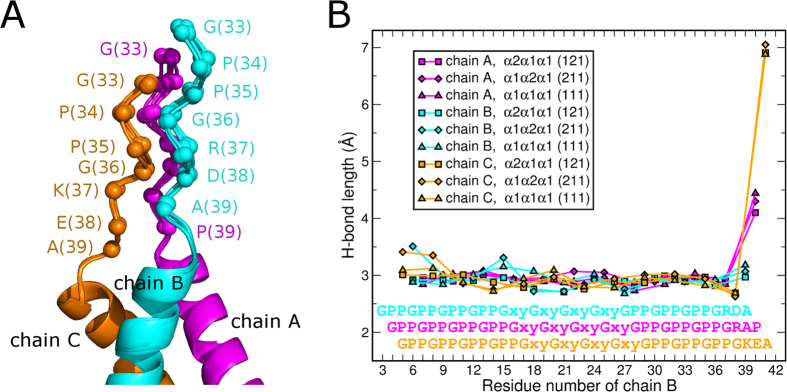
Triple helix – NC2 domain interface. (**A**) Four structures (121nat, 121sm, 111sm and 211sm) are superimposed within the NC2 domain core. C_α_-positions of residues 33–39 are shown as spheres. (**B**) Inter-chain hydrogen bond lengths within the triple-helix and the interface. Chain A (α1 of NC2) – magenta, chain B (α2 of NC2) – cyan, chain C (α3 of NC2) – dark orange.

The overall structure of the triple helical sequences is typical for the structure of a triple helix. Despite the variations of composition (homo-trimeric for 111) and stagger (121 or 211) that might not represent a native stagger, the regions of sequences derived from type I collagen are well structured. A set of unique side chain interactions is observed for each composition (Fig. 3A). The most important observation is that the triple-helical chain stagger is entirely determined by the NC2 domain (Fig. 5A). Namely, a triple helical chain linked to chain B of the NC2 domain is always in the leading position, the one linked to A is in the middle, and the third one linked to C is in the trailing position. We suggest here to use a rule of BAC-translation: chain B is leading, A is middle, C is trailing. This way, construct 121 (or α1_A_α2_B_α1 _C_) translates into staggering order of α2α1α1, whereas 211 translates into α1α2α1.

### Interface between the triple helix and trimerization domains

The right-handed bundle of α helices (not a very common structure) of the NC2 domain congruently continues into the right-handed superhelix of the collagenous part. Visual analysis of the interface region suggests a broadening of the triple helical end before the NC2 domain starts. To analyze and quantify the opening of the triple helix and transition into the NC2 domain we identified and plotted the ladder of recurrent N–H_(G)_…O = C_(X)_ hydrogen bonds (characteristic collagenous bonds between glycine in one chain and an amino acid in X position of an adjacent chain) that form within the triple helix and the beginning of the NC2 domain (Fig. 5B). Remarkably, no opening was identified within the host-guest collagen peptide (GPP)_4_-(GXY)_4_-(GPP)_3_ sequences linked to the NC2 domain. Moreover, first glycine residues that were originally assigned to the beginning of the NC2 domain are still part of the triple helix without any sign of disturbance. Even an alanine residue (Ala39, +3 position from the first glycine) in the leading chain (chain B) does still form a reliable hydrogen bond with lysine 37 in the trailing chain (chain C) (Fig. 5B).

**Figure 6:**
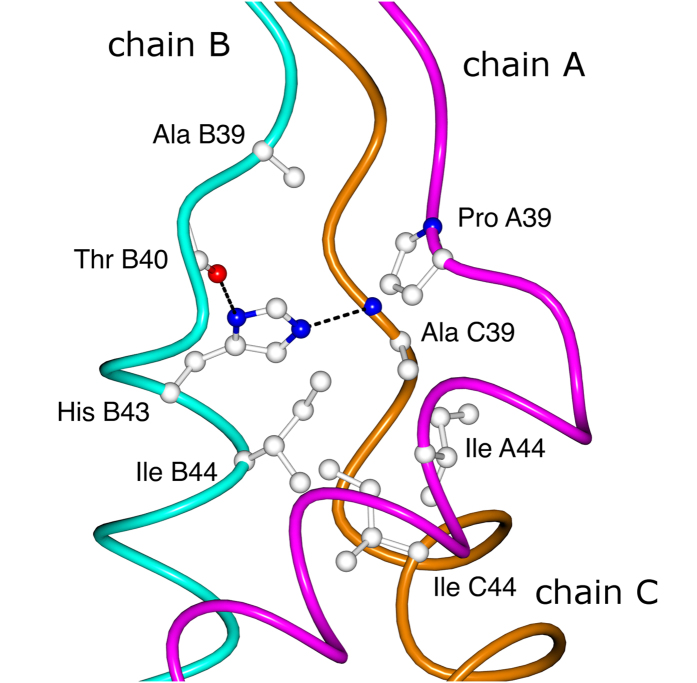
The core residues at the interface between the triple-helix and the NC2 domain (shown 121sm). Chain A (α1 of NC2) – magenta, chain B (α2 of NC2) – cyan, chain C (α3 of NC2) – dark orange.

The actual opening of the “triple helix” happens only at already the non-collagenous sequence of the NC2 domain (Fig. 6), where residues such as Ala39, Thr40, His43 of chain B, Pro39 of chain A and Ala39 of chain C, are the capping residues of the hydrophobic core of the NC2 domain (starting from Ile44). In other words these capping residues constitute a pyramid that connects a “zero” hydrophobic core (formed by glycines) of the triple helix to the real hydrophobic core of the NC2 domain. Interestingly, whereas His43 of chain B is involved in the intra- (Thr40, chain B) and inter-chain (Ala39, chain C) hydrogen bonding within the capping core (Fig. 6), solvent exposed His43 of chain A is interfacing with Asp41 of chain B (Fig. 4), further emphasizing the asymmetric nature of collagen.

## Discussion

Collagen is the most plentiful protein in our body fulfilling structural and biologically active roles in multiple physiological processes as well as in pathology. Numerous heritable and acquired diseases are associated with collagen. Atherosclerosis, fibrosis, osteoarthritis, rheumatoid arthritis, diabetes, cancer are just few diseases where collagen function is adversely affected. 28 collagen types are formed from polypeptides encoded by 42 distinct genes, frequently in several isoforms. In addition, more than 20 additional proteins adopt collagen-like structures such as collectins, ficolins, and scavenger receptors^26^. Our knowledge of structural and functional organization of this universe is very fragmented and limited to just few homo-trimeric collagenous fragments and some non-collagenous domains. The only example where a triple-helix has been crystallized with an adjacent non-triple-helical domain is the structure of the (GPP)_10_-foldon construct^27^. Foldon, a trimeric nucleation domain for a classical coiled coil, leads to a severe kink and disturbance of the triple helix attached to it. Until now there was no robust method to produce fragments of hetero-trimeric collagenous regions; this has significantly limited the repertoire of reagents that are available to study the role of collagens in development, remodeling and cell signaling.

As revealed by the structural analysis of the host-guest system reported here, such a method is now available. A collagenous sequence connected to the α2 chain of the NC2 domain will have the leading position, whereas collagenous sequences linked to α1 and α3 chains will be in the middle and trailing positions, respectively. To avoid confusion we suggest to label α1, α2 and α3 chains of the NC2 domain as chains A, B and C. Connecting collagenous sequences to respectively B, A and C chains will place them in the leading, middle and trailing positions (the BAC translation rule). The small size of the NC2 domain and the ability to recombinantly express individual chains in bacteria and later assemble them *in vitro* make this system easily adoptable in any laboratory with basic molecular biology techniques. If needed the expression system can be transferred to eukaryotic cells to obtain certain post-translational modifications of proline and lysine residues. Moreover a peptide synthesis with specifically modified residues is still possible for these sizes.

Crystal structures of 111, 121 and 211 constructs demonstrated that a staggering order can be manipulated at least for short native collagenous sequences, meaning that such sequences are rather adaptive to various abnormal conformations. Nevertheless, these alternative conformations demonstrated differences in thermal stability and affinity to a ligand^23^. Namely, we have shown previously that the 112 construct (with the α1α1α2 staggering order of the triple helical portion of type I collagen) had the highest binding affinity to von Willebrand factor A3 domain, and highest thermal stability of the different constructs. If the high binding affinity and high thermal stability is indicative, then the stagger of type I collagen is α1 chain in the leading position, α1 chain in the middle position and the α2 chain in the trailing position. Further experiments with other fragments of type I collagen as well as other hetero-trimeric types of collagen are anticipated to clarify this general problem.

At least for type IX collagen we can conclude now that the stagger of the central collagenous domain (COL2) is α2α1α3 and it is determined by the NC2 domain. Staggers and its staggering mechanisms of other collagenous domains in type IX and other collagens remain to be elucidated. The most intriguing structural studies would include a junctional region between a triple-helical and trimerization domain in such hetero-trimeric collagens as type I and IV, as well as in hetero-trimeric collagen-related complement protein C1q.

In summary, these data detail the structural organization of the triple-helix- to- trimerization domain interface of type IX collagen and the mechanism of staggering. The current constructs provide a straightforward tool to produce any collagen fragments of interest with a controlled composition and stagger.

## Methods Summary

All constructs were expressed, accordingly assembled and purified as described^23^. Only polypeptides containing the α1 chain of the NC2 domain (chain A) were labeled with selenomethionine for phasing using methionine-deficient *E. coli* strain B834 (DE3) and a medium composed of SelenoMet Medium Base and SelenoMet Nutrient Mix (Athena Enzyme Systems).

The complexes were crystallized by vapor diffusion with the following crystallization conditions:

111sm: 0.1 M BisTris pH 6.4, 14% PEG MME 5,000 + 20% glycerol (cryo)

121nat and 121sm: 0.1 M HEPES pH 7.5, 50 mM Na-Acetate, 17% PEG 3,350 + 20% glycerol (cryo)

211sm: 0.1 M BisTris pH 6.0, 16% PEG MME 5,000 + 20% glycerol (cryo).

Diffraction data were collected at the Advanced Light Source beamline 4.2.2. The diffraction images were indexed, integrated, and scaled using HKL2000^28^. Selenomethionine crystals were used for a three-wavelength MAD data collection procedure. The program PHENIX^29^ was used for the determination of Se atom positions, phasing, density modifications, and automatic building of partial models. Iterative cycles of model extension/correction and refinement were performed using the programs COOT^30^ and PHENIX^29^, respectively. A model from the selenomethionine crystal of 121sm was directly used for the refinement of native structure 121nat.

Crystal diffraction data, phasing and refinement statistics are presented in Table 1.

## Additional Information

**Accession codes:** Atomic coordinates and structure factor amplitudes have been deposited in the Protein Data Bank under accession numbers 5CTD, 5CTI, 5CVA and 5CVB.

**How to cite this article**: Boudko, S. P. and Bächinger, H. P. Structural insight for chain selection and stagger control in collagen. *Sci. Rep.* **6**, 37831; doi: 10.1038/srep37831 (2016).

**Publisher's note:** Springer Nature remains neutral with regard to jurisdictional claims in published maps and institutional affiliations.

## Figures and Tables

**Table 1:** Data collection, phasing and refinement statistics for native and MAD (SeMet) structures.

	121nat(native)(PDB ID: 5CTI)	121sm(MAD) (PDB ID: 5CTD)			211sm(MAD) (PDB ID: 5CVA)			111sm(MAD) (PDB ID: 5CVB)		
**Data collection**										
Space group	P2_1_	P2_1_			P2_1_			P2_1_		
Cell dimensions										
*a, b, c* (Å)	27.78, 55.91, 61.66	27.89, 55.92, 62.10			52.41, 63.98, 65.37			51.77, 64.46, 65.39		
α, β, γ (°)	90.00, 92.35, 90.00	90.00, 92.86, 90.00			90.00, 112.75, 90.00			90.00, 112.72, 90.00		
		*Peak*	*Inflection*	*Remote*	*Peak*	*Inflection*	*Remote*	*Peak*	*Inflection*	*Remote*
Wavelength	1.5418	0.97872	0.97918	0.96487	0.97879	0.97901	0.96337	0.97866	0.97887	0.96338
Resolution (Å)	50.00–1.90 (1.93–1.90)*	50.00–1.60 (1.63–1.60)			50.00–2.10 (2.14–2.10)			50.0–2.25 (2.29–2.25)		
*R*_merge_	0.068 (0.419)	0.084 (0.360)	0.075 (0.380)	0.076 (0.375)	0.094 (0.286)	0.090 (0.266)	0.089 (0.275)	0.115 (0.363)	0.103 (0.330)	0.096 (0.301)
*I*/σ*I*	15.3 (1.7)	11.85 (2.96)	11.45 (2.63)	10.96 (2.39)	14.86 (3.19)	14.94 (3.27)	14.93 (3.12)	12.89 (2.89)	12.74 (3.24)	10.08 (2.53)
Completeness (%)	99.1 (94.7)	96.7 (88.3)	96.4 (84.3)	96.4 (83.2)	89.9 (48.9)	88.1 (43.0)	88.8 (45.4)	92.6 (54.7)	94.8 (63.6)	91.5 (52.1)
Redundancy	11.9 (4.0)	3.8 (3.0)	3.8 (2.9)	3.8 (2.8)	6.2 (3.5)	6.3 (3.3)	6.6 (3.5)	6.7 (3.6)	5.3 (3.4)	3.5 (2.5)
**Refinement**										
Resolution (Å)	30.80–1.90 (1.96–1.90)	41.53–1.60 (1.64–1.60)			48.33–2.10 (2.15–2.10)			47.75–2.25 (2.32–2.25)		
No. reflections	14836		23880			20036			16750	
*R*_work_/*R*_free_	0.160/0.199 (0.220/0.278)	0.177/0.203 (0.271/0.271)			0.238/0.279 (0.304/0.368)			0.227/0.278 (0.267/0.389)		
No. atoms	3082		3061			2572			2786	
Protein	2895		2867			2484			2660	
Ligand/ion	42		0			12			30	
Water	145		194			76			96	
*B*-factors	26.68		26.89			45.24			43.90	
Protein	26.10		26.22			45.41			44.11	
Ligand/ion	44.43		-			42.22			41.39	
Water	33.06		36.74			39.97			38.73	
R.m.s deviations										
Bond lengths (Å)	0.011		0.012			0.006			0.005	
Bond angles (°)	1.456		1.383			0.986			1.153	

*Values in parentheses are for highest-resolution shell.
